# Supplementary material for: Associations between blood type and COVID-19 infection, intubation, and death
Source: Nat Commun. 2020 Nov 13;11:5761. doi: 10.1038/s41467-020-19623-x (PMC7666188; doi:10.1038/s41467-020-19623-x)
Supplement: Supplementary file 1 — Supplementary Information [file 41467_2020_19623_MOESM1_ESM.pdf]

Supplemental information

Supplementary Figure 1: **Flow diagram of inclusion and exclusion criteria for the cohort used.** Numbers indicate the number of patients in each group. Groups in the right column were excluded.

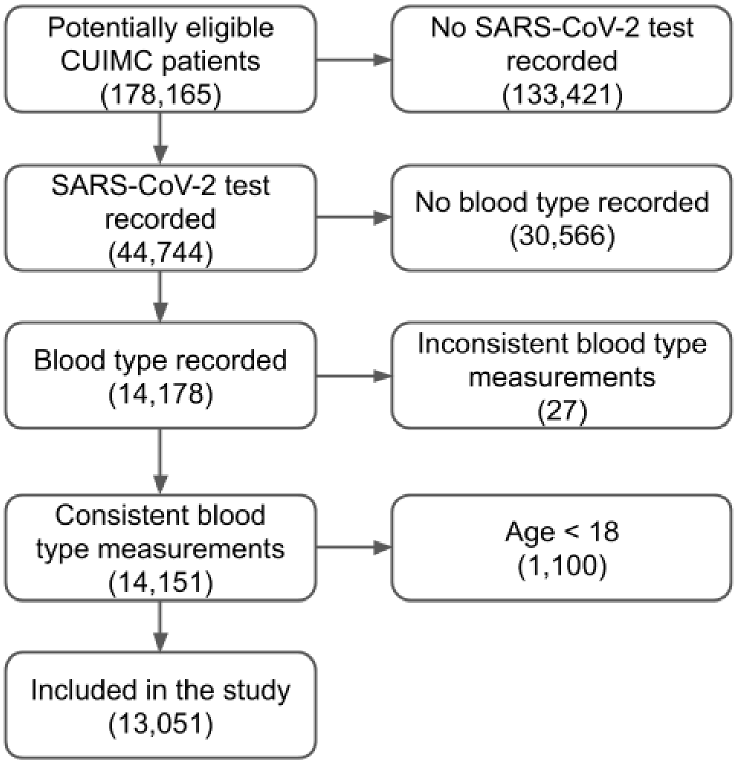

Supplementary Figure 2: **Graphical model of the system under investigation.** We sought to estimate the total effects of blood type on the COVID-19 outcomes under investigation. Confounding can be controlled by adjusting for ancestry. Selection bias cannot be controlled fully, and as a result, our estimates are conditional on presentation to the hospital during the COVID-19 pandemic.

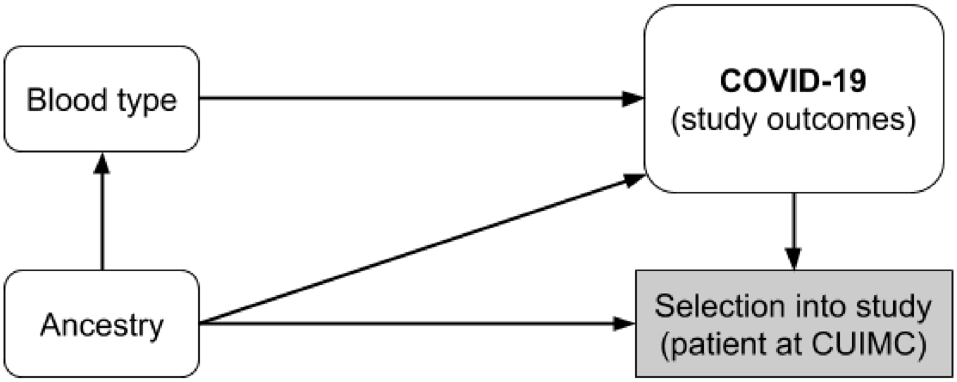

Supplementary Table 1: **Chi-squared tests to evaluate dependence between blood type and having received a test for SARS-CoV-2**. ABO had three degrees of freedom, while Rh(D) had one degree of freedom.

Blood group	SARS-CoV-2 tested	non-SARS-CoV-2-tested	Chi-squared	p-value
ABO	A: 4298 (32.9%), AB: 559 (4.3%), B: 2033 (15.6%), O: 6161 (47.2%)	A: 34156 (32.7%), AB: 4405 (4.2%), B: 15590 (14.9%), O: 50305 (48.2%)	5.79	0.122
Rh(D)	neg: 1195 (9.2%), pos: 11856 (90.8%)	neg: 9644 (9.2%), pos: 94812 (90.8%)	0.0716	0.789
