# Supplementary material for: Associations between blood type and COVID-19 infection, intubation, and death
Source: Nat Commun. 2020 Nov 13;11:5761. doi: 10.1038/s41467-020-19623-x (PMC7666188; doi:10.1038/s41467-020-19623-x)
Supplement: Supplementary file 2 — Reporting Summary [file 41467_2020_19623_MOESM2_ESM.pdf]

nature research

Corresponding author(s): Dr. Nicholas Tatonetti

Last updated by author(s): October 8, 2020

Reporting Summary

Nature Research wishes to improve the reproducibility of the work that we publish. This form provides structure for consistency and transparency in reporting. For further information on Nature Research policies, see our Editorial Policies and the Editorial Policy Checklist.

Statistics

For all statistical analyses, confirm that the following items are present in the figure legend, table legend, main text, or Methods section.

n/a	Confirmed	
☐	☒	The exact sample size (*n*) for each experimental group/condition, given as a discrete number and unit of measurement
☒	☐	A statement on whether measurements were taken from distinct samples or whether the same sample was measured repeatedly
☒	☐	The statistical test(s) used AND whether they are one- or two-sided<br>*Only common tests should be described solely by name; describe more complex techniques in the Methods section.*
☐	☒	A description of all covariates tested
☐	☒	A description of any assumptions or corrections, such as tests of normality and adjustment for multiple comparisons
☐	☒	A full description of the statistical parameters including central tendency (e.g. means) or other basic estimates (e.g. regression coefficient) AND variation (e.g. standard deviation) or associated estimates of uncertainty (e.g. confidence intervals)
☒	☐	For null hypothesis testing, the test statistic (e.g. *F*, *t*, *r*) with confidence intervals, effect sizes, degrees of freedom and *P* value noted<br>*Give P values as exact values whenever suitable.*
☒	☐	For Bayesian analysis, information on the choice of priors and Markov chain Monte Carlo settings
☒	☐	For hierarchical and complex designs, identification of the appropriate level for tests and full reporting of outcomes
☐	☒	Estimates of effect sizes (e.g. Cohen's *d*, Pearson's *r*), indicating how they were calculated

Our web collection on statistics for biologists contains articles on many of the points above.

Software and code

Policy information about availability of computer code

Data collection	We used MySQL version 5.6, R version 4.0.1, tidyverse meta-package version 1.1.2. Code used for data collection is available at https://github.com/zietzm/abo_covid_analysis.
Data analysis	We used MySQL version 5.6, R version 4.0.1, tidyverse meta-package version 1.1.2. Code used for data analysis is available at https://github.com/zietzm/abo_covid_analysis. We also used the Manubot software for drafting the manuscript (https://github.com/zietzm/abo_covid).

For manuscripts utilizing custom algorithms or software that are central to the research but not yet described in published literature, software must be made available to editors and reviewers. We strongly encourage code deposition in a community repository (e.g. GitHub). See the Nature Research guidelines for submitting code & software for further information.

Data

Policy information about availability of data

All manuscripts must include a data availability statement. This statement should provide the following information, where applicable:

- Accession codes, unique identifiers, or web links for publicly available datasets
- A list of figures that have associated raw data
- A description of any restrictions on data availability

Our results are based on individual-level patient data from non publicly accessible electronic medical records. Current institutional policy prohibits release of individual-level data to protect patient privacy. In addition, these data are legally protected in the United States against public release, pursuant to 1996 Public Law 104-191 (HIPAA). Aggregate-level data for all fields we considered are available in table 1.

Field-specific reporting

Please select the one below that is the best fit for your research. If you are not sure, read the appropriate sections before making your selection.

☒ Life sciences ☐ Behavioural & social sciences ☐ Ecological, evolutionary & environmental sciences

For a reference copy of the document with all sections, see nature.com/documents/nr-reporting-summary-flat.pdf

Life sciences study design

All studies must disclose on these points even when the disclosure is negative.

Sample size	Sample size was every patient at NYP/CUIMC who met the eligibility criteria. Sample sizes were larger than most comparable studies, and our estimates were largely consistent.
Data exclusions	We excluded individuals with contradictory blood type measurements (likely errors), individuals who didn't receive any tests for SARS-CoV-2 (not relevant to the study), and children (disproportionately low risk due to COVID-19, high risk of incorrect associations between outcomes and COVID-19). The criteria for inclusion and exclusion were established before data were considered.
Replication	We evaluated three different measures of COVID-19 severity (tested positive in the hospital, intubated, died). While not replicated across different sites, these are approximate variations on the same outcome.
Randomization	As blood type is genetically determined, the treatments could not be randomized in this study. We made adjustments for race and ethnicity, proxies for ancestry, the relevant confounder for this study.
Blinding	Researchers were only blinded to the identity of identifying patient information as required by law. Group allocations are integral information to the analysis, which could not have been conducted under additional blinding.

Reporting for specific materials, systems and methods

We require information from authors about some types of materials, experimental systems and methods used in many studies. Here, indicate whether each material, system or method listed is relevant to your study. If you are not sure if a list item applies to your research, read the appropriate section before selecting a response.

Materials & experimental systems

n/a	Involved in the study	
☒	☐	Antibodies
☒	☐	Eukaryotic cell lines
☒	☐	Palaeontology and archaeology
☒	☐	Animals and other organisms
☐	☒	Human research participants
☒	☐	Clinical data
☒	☐	Dual use research of concern

Methods

n/a	Involved in the study	
☒	☐	ChIP-seq
☒	☐	Flow cytometry
☒	☐	MRI-based neuroimaging

Human research participants

Policy information about studies involving human research participants

Population characteristics	This information is provided in Table 1 of the manuscript.
Recruitment	No recruitment was necessary for this study. Patients were included who visited NYP/CUIMC between March 1 and August 1, 2020 for personal medical reasons, not on account of the current study.
Ethics oversight	This study was approved by the Columbia University IRB under #AAAL0601.

Note that full information on the approval of the study protocol must also be provided in the manuscript.
